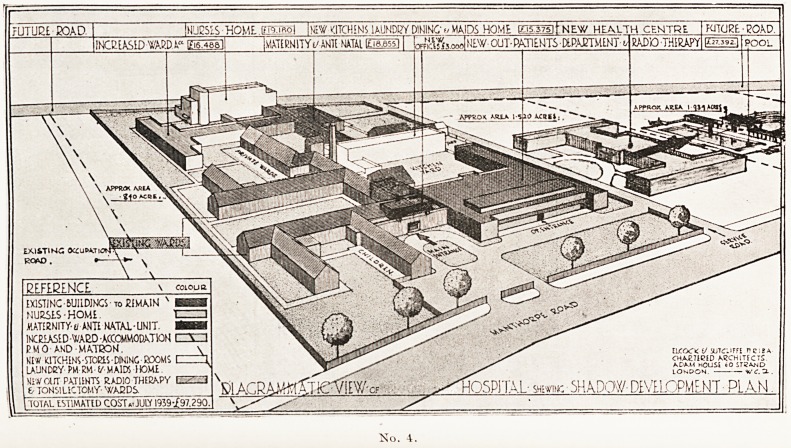# The Future of Health and Hospital Services from an Architect's Point of View

**Published:** 1943

**Authors:** Charles E. Elcock


### The Bristol Medico-Chirurgical Journal

"*Scire est nescire, nisi id me*
*Scire alius sciret.*"

AUTUMN, 1943.

### THE FUTURE OF HEALTH AND HOSPITAL SERVICES FROM AN ARCHITECT'S POINT OF VIEW.

BY

Charles E. Elcock, F.R.I.B.A.

(An Address given at Bristol, on November 27th, 1942).

You have honoured me by asking me to speak on "Modern Health Services" as the architect sees them. It would be a poor return if I merely made general remarks and said that "everything in the garden was lovely." It is probable that I may throw some bricks about and I may tread on many important toes and local prejudices. But I come to give you my very modest knowledge on the subject untrammelled by any local opinions or prejudices. If in any way I should be able to assist you in the formulation of a scheme worthy of your ancient city and of this very important district, I shall be more than rewarded; and if anything seems very controversial, please put it down to that proverbial coat-trailing propensity which appears to be associated with my exuberant Hibernian ancestry.

In many people's minds the work of the architect is limited to a pretty sketch or two and some nicely coloured plans. There is little realization that many working drawings are required, very carefully figured and showing every detail of the work. In a building like the *Daily Telegraph* offices in Fleet Street over 3,000 drawings were prepared in my office. But there is more in an architect's method than making dirty lines on clean paper. If he is to be a real creator, he must understand quite clearly the needs of his clients, both the needs of the individual client and the needs of the community. To do this he must keep his finger on the throbbing pulse of national and community life, and its needs and its wants. He must saturate his mind with fine things in art, in music, in literature. He must thoroughly understand his architectural history and construction, and in addition he must study social life and its history, and then—he must put it all away into the treasure-house of his soul and out of a cultured and well-stored mind bring out of his treasure-house things new and old. But particularly new, for they must be touched with his own personality, which must ever be renewed, and so his creative work will be vitally alive. He may be criticized, he may be ostracized, but he will never be sterilized. So the architect coming to your great city, crammed full of history, romance, and the adventure of your great merchants, pullulating with a distinctive life of its own, feels again that in the wrecked streets and buildings of your city you have an opportunity of re-creation which has called to the forward-looking minds of your citizens with somewhat the same feelings of your great ancestors, who in the virgin lands and deserts across the great oceans saw visions of great opportunities and noble futures. So out of the desert of your wrecked city you are thinking and planning for your sons and daughters, for your grandsons and granddaughters, a fairer city than Bristol has ever known, retaining inviolate the soul of its people and all the historic associations in which this city is steeped. People are more important than buildings, and what goes on in buildings is more important than their outer husks.

Whatever plans are therefore made for the city they must not be little in scope and vision. There is no magic in littleness to stir the people's blood. It has been greatly said that "without vision the people perish," or, as Stephenson said, "many a man goes to sleep between the chimney pots and the telegraph wires." Between the comfort of the home fireside—(subject, of course, to the Fuel Controller !) and the claims of business, we must *not*—if we are to be worthy—allow these things to dull our vision of that great city which must arise out of the ruin which now exists. We must not be like Bunyan's "man with the muckrake," who kept on scrabbling about in the dirt, the scraping of his muckrake drowning the beating of the angel's pinions hovering over him—"an angel bearing a crown." Therefore your plans must be big, full of vision, perhaps only realizable bit by bit, but all related to the great conception of the whole. The whole thing full of the light in a great people's eyes, and crammed with the fire of the creative spirit of God. The humblest dweller in the smallest street, equally with the great merchant, must feel some sparkle of this starry vision.

Amongst the planning of more immediate activities must come the provision for the health of the community. To many people this may appear to consist of the provision of better and more co-ordinated hospitals with their clinical and other services. In Bristol you have many hospitals which have to some extent been haphazardly imposed on the city without co-ordination, and with many amenities which might possibly have been *collected into one central building.* It may be that in planning for the future, existing and new hospitals under a co-ordinating Hospital Council will be arranged in close proximity to a New Hospital Centre convenient for medical students and contiguous or near to the University Medical School. With a central buying and supply department, and a central laundry for all the hospitals in the district, considerable economy might be effected both in administrative and staff costs, and in tenders for supplies for all hospitals in the area of the Divisional Council.

You have probably noted already that in a triangle which has a base approximately 490 yards long, and a height from base to apex of, say, 140 yards, there are five hospitals in central Bristol. The distance from the University Medical School to the centre of this triangle being, say, 300 yards, makes for the obvious consideration of a site in this area as being the possible site of any Medical Centre, if such was thought an essential part of the future hospital service. This Hospital centre, whilst having the usual special hospitals and their services closely planned and related, and including also a psychiatric centre for the study of incipient and acute mental cases, should also have as one of its main features a special centre for the study of preventive medicine and social hygiene, with its field service for extra-mural work and its essential research laboratories which would serve the district covered by the co-ordinating Hospital Council.

I do not know what is the actual area served by the Bristol and District Divisional Hospitals Council, but it will certainly include both urban and rural areas with very diversified needs and with hospitals both large and small which may be modern and well-equipped, or the reverse. It would, therefore, be an essential and urgent feature of any future activity that survey should be made of the area or district and a detailed report made from the point of view of the doctor, the nurse and the patient. This would require a technical survey by competent medical and surgical representatives, and a survey of each hospital by an architect—an expert in hospital planning. A hospital accountant would also be required to report on the accounting methods of each hospital and its financial standing. An experienced and competent medical-social worker would also prepare a social survey of industrial and living conditions in the district.

By collecting and collating all these reports a scientific study of the existing hospital services would be at hand with the necessary data to enable a select Committee to draft a final comprehensive report on the present and future Health Services of the agreed Bristol and District Division. Special notice would be taken of the social conditions of each area within the district and the report would particularly include a statement as to the preventive and social hygiene work at present undertaken in the district. Based on the report certain new hospitals might be necessary, with possible rearrangement of existing hospitals. A further development might consist in enlarging some of the rural hospitals so that they might also act as convalescent hospitals for the Urban areas, as well as catering for the acute sick in their immediate neighbourhood.

As economy in material and cost will be an immediate problem in post-war building, it will be necessary for the architect to be willing to strike out on new lines. There must be no complacent acceptance of old traditions. In architecture complacency and tradition make bad masters. The stones they build with have had time to gather the proverbial moss. But there is a most extraordinary divergence in the cost of both large and small hospitals. For instance, a large voluntary hospital lately erected in London has cost over £2,000 a bed. This is comparable with a somewhat similar hospital lately erected in Lancashire which cost under £700 a bed. If allowance is made for certain special features in the London Hospital, it is still impossible to agree to a figure of over £2,000 a bed. A small Wiltshire Cottage Hospital erected complete at a cost of under £500 a bed has to be compared with a Yorkshire Cottage Hospital of exactly the same size costing £1,000 a bed. This extraordinary variation in cost shows that there is a need for some standardization of requirements as regards accommodation, construction and equipment. The definite essentials of a modern hospital should be agreed on by conference between the Medical and Nursing Administrative Staffs in co-operation with architects skilled in hospital planning. A great deal remains to be done in the development of modern hospital planning, as from 1856 to, say, 1914, hospital planning—with few exceptions—became standardized, crystallized and even fossilized. The large hospitals of the future must be as clean and scientific in design as a surgeon's lancet. They must partake more of the nature of "factories for health" rather than offering an imposing opportunity for architectural display. As far as possible corridors must be eliminated, sanitary services must be collected together, noise must be lessened, and, above all, there must be a form of plan and construction which will allow for the utmost flexibility in rearrangement of rooms and walls so as to keep abreast of new developments in medical and surgical requirements. By the adoption of what I have styled the "health factory" type of plan, with its scientific clarity and flexibility of design, and its elimination of outmoded and unnecessary features, not only is capital outlay saved but large economies can be effected in annual administrative costs. I have actually worked out a plan of a General Hospital for 500 patients on this new system of planning, and have had the costs properly estimated. The economy is at once apparent when the figures show that this General Hospital fully

A-CONTINUOUS-VERANDA-WARD-HOSPITAL-TO-ACCOMMODATE-480-BEDS

NURSES AND MAIDS-HOME

ROADWAY

ENTRANCE COURT

MORTUARY

OPERATING

ISOLATION

THE-WARD-UNITS

N

BIRDS-EYE-VIEW-FROM-THE-SOUTH-EAST- [DIAGRAMMATIC]

CHARLES-E-ELCOCK-F.R.I.B.A.

equipped with all fixed fittings—but without loose furniture and not including a medical school, but inclusive of a Nurses' Home and heating and electric installations could be erected in this country at 1939 prices for under £600 a bed. Allowing for a possible one-third increase in post-war costs, it still shows a remarkable economy in construction.

Here Mr. Elcock illustrated his lecture with lantern slides, four of which, by the courtesy of the Royal Society of Medicine, we are permitted to reproduce, together with extracts from a paper printed in the *Proceedings of the Society* (March, 1942).

No. 1 shows the new type of Veranda-ward which Mr. Elcock first designed in his plans for the extension of the Hertford County Hospital. The old-pattern wards had walls of which 70 per cent. of the surface was filled with bricks while the window openings formed only 30 per cent. He reversed these proportions, turning the wall space into long windows which could slide open and convert the ward into a veranda. The beds grouped between glass screens are placed parallel with the wall instead of at right angles to it.

Nos. 2 and 3 are designs for a " Health Factory." This consists diagrammatically of a long hall 20 feet wide placed with its axis east and west. Large folding windows come between the supporting stanchions on the south façade. A typical floor would be divided into units of thirty beds each, and might be three or six units long and from three to ten floors high, depending upon the number of beds required. In the centre of each unit on the northern side are placed the necessary subsidiary rooms, toilet rooms and utility services. Each unit is separated from the adjacent units by a cross wall, but communicates through the escape staircase lobby. Each utility wing on the north side has its own bed and passenger elevators and staircase, but there is no connecting corridor except on the ground floor. All internal walls can be removed without interference with the structure, which allows the utmost flexibility for future needs. All sanitary and main service pipes are in the northern service wings. The main structure would be of steel with thin external walls lined with non-conducting material and noise proof floors, sanitary apparatus, piping and doors.

" No. 4 is an actual project for expanding a hospital into the complete Health Centre " of a large provincial town of the future. The centre is to consist of the main hospital, a new nurses' home, kitchens, out-patients departments, maternity unit, modern research laboratories, but its chief feature will be preventive and educative therapy in the form of clinics, lecture halls and class rooms, swimming bath, outdoor and indoor gymnasia and a variety of other amenities all leading to the enthusiastic teaching of how to live healthfully both young and old. And this is only a small part of the whole " Health City of the Future " which Mr. Elcock envisages, with its parks, swimming baths, gymnasia, schools, community halls, hobby and craft rooms with its museums and lecture halls in which people can be taught how to eat and drink, how to dress and how to employ their leisure. Thus, instead of health being associated with disease, it will be associated with healthy living.

And yet—after all—we have only touched the negative side of the subject of future health services—the curing of established disease or injury.

*The great positive future of our health services is the prevention of disease and the teaching of people how to live happy and healthy-minded lives.*

How to work healthily—how to enjoy their leisure healthily—how to keep clean, what to eat and how to cook it—and so eventually —*to keep out of the hospital.* The whole subject of the health of the people has for far too long been limited to caring for the sick, to relieving or curing the diseased or injured.

As an architect who has been responsible for the planning of many types of hospitals and who has studied these buildings in Europe and America by personal visits, I feel that the time has certainly come when a new view of our community health services is demanded. I therefore suggest as a layman—that the next great advance in medical practice will be the prevention of disease and the teaching of the principles of healthy living. We will always have the necessity for the provision of hospitals and their appurtenances for the care of those actually struck down by accident or disease, but the time is fully ripe for a different outlook and for the dissemination of the facts which make for a healthy mind in a healthy body. We must cease to think merely of hospitals as places to educate young doctors, instruct nurses, inoculate guinea-pigs, or as elaborate plumbing depots—they must be part of the educational facilities of a community, where education in healthful living is actually the mainspring of their activities.

I visualize these health centres as more than Community Centres, but they would include these. I can see—diagrammatically—a large campus or place having at one end its Municipal Offices and Council Chambers, and the usual concomitants of these buildings. Along two sides would be the Art Gallery and Museums, the Library, and the Public Hall fitted for concerts and dramatic performances and other public uses. On the other side might be the School of Art and the Technical College. At the other end would be the Community Centre, containing the very important Reception and Enquiry departments, where new people coming to the town could be at once advised as to the places of residence—work possibilities—where to shop; and incidentally, be initiated into the life of the city, town, or village, and feel they had come amongst friends. A large central lounge with various quiet and noisy rooms with suitable refreshment facilities would also be an important feature. There would be rooms for Adult Educational activities, hobby and craft rooms to enable people who have not the necessary space in their smaller houses to make things for themselves, gymnasia for juniors and adults. In addition there would be departments devoted to bodily health. Maternity *and Paternity* and Child Welfare Clinics,

The Continuous-Veranda Ward Hospital.

Charles E. Elcock F.R.I.B.A.

and Examination and Consulting Rooms for regular health surveys and advice. All these would be linked closely with the public health services, the clinics and hospitals, and the Field Service Health Unit for the Divisional District. This Field Service Unit would be centrally situated—possibly at the Medical Centre—with its own research laboratories and a staff of physicians, nurses and social workers, ready to go anywhere in the district to take charge in case of industrial disaster, or assist or investigate the outbreak of disease in the district.

I would suggest that the development of these health centres is something bigger than the provision of mere "health factories"—health houses or hospitals. To me the wire fence round the cliff top is more interesting than the more spectacular ambulance at the bottom.

Personally, I am getting somewhat tired of the insistence on curing disease. I want there to be very little disease to cure. I visualize the abounding health and happiness of the people as becoming the greatest future glory of civic life.

We have accustomed ourselves to point with pride to our huge medical centres and hospitals, quite forgetting that the greater these are, the greater is the shame that such things should be required. I can see a city with its parks, swimming baths, gymnasia, schools, community halls, hobby and craft rooms, with its museums and lecture halls, in which people can be taught how to eat and drink, how to dress, how to employ their leisure: in short, how to live rightly; and all these linked up closely and intimately with preventive health clinics of all sorts—infantile, adolescent and adult, and round the corner, somewhat ashamed of its existence, the hospital, to take all those unfortunates whom accident or disease has laid low. Instead of health becoming associated with disease, it will thus become associated with healthy living.

Going through the streets of our cities and small towns we see many people standing about at corners, lounging in bars, hump-shouldered and shabby, and in an ignorant effort to pass time away spending their money on "that which is not bread." Go through the West End of our cities into our well-established clubs and our precariously established night-clubs and see the lounging men and women, who are presumably better educated, doing exactly the same thing. Neither their physique nor their conversation shows culture of body or of mind. We want to restore to humanity some of its innate dignity and an educated sense of its worth.

There are obviously very important details to be discussed—the obsolete arrangements of honorary medical staffs—the admission of private practitioners to attend to their own patients in hospitals—the position of specialists—the training of medical students—the salaries, training and housing of the registered qualified nursing staffs—the status and duties of hospital administrators—the selection of Members of Voluntary Hospital Boards—the representation on State and County Hospital Boards of Divisional Council Representatives. In addition there will be the details of the types of Hospitals — Convalescent Homes — Specialist Hospitals — Medical Centres and Sanatoria, and Community Health Centres and Clinics. These matters must be based on the Surveys and Reports from the District Divisional Council, but sufficient has been stated to indicate the magnitude of the task which lies before any body of men and women who wish to undertake a serious study of future health services for their district. Above all—it is the Health of the People you are to consider and not merely disease-curing.

The matter is urgent. Men are fighting now in the air, on the sea, and on land so that they may come back to their country and find something better in the future. They will be in no mood to be satisfied with "Homes for Heroes" which do not materialize, nor petty party rearrangements of social and economic conditions. They will rightly demand that this unparalleled sacrifice of life and treasure should eventuate in well-drawn and practical propositions on which they can be at once employed. Only by immediate initiation of surveys and the preparation of workable schemes can we check any natural outburst of disappointment with all its obvious and awkward possibilities.

In this connection the words of Sir William Beveridge in an address delivered in London last week, are worthy of study. He said : "A major contribution to victory would be to give all on war service—whether in the Forces or in civilian work—confidence that the Government had effective plans for maintaining employment after the war, and would use all the powers of the State, so far as necessary, for that purpose.

"It is not possible to trust to the methods of the 'last peace'—of private enterprise without national planning—to bring about the necessary readjustment of our productive effort in the difficult transition period after the war. National planning is essential however the plan is executed. But it is vital to preserve initiative, and enterprise. The practical problem is that of discovering how to combine the proved benefits of private enterprise at private risk in the past with the necessity of national planning in the aftermath of war. The solution can be found only by thorough unbiased investigation and discussion now."

Therefore do not forget the urgency of the work which lies before you. The Survey and collation of facts, the preparation of a sound working scheme of this magnitude is a matter of really serious and detailed work. A time limit should be set and the report should be issued by a certain date, or else your "Great Plan" may remain vague, visionary and nebulous. Get the whole people of your Region or District enthused with "The Big Idea," ask them to sit at your local enquiries and let them recapture their youth in helping forward the health and happiness of their own generation and of the generations to come.

### QUESTIONS AND ANSWERS.

*Q.* MR. J. NELSON MEREDITH, F.R.I.B.A. : Could Mr. Elcock give his views on underground operating theatres ?

*A.* MR. ELCOCK : I do not think there is any difficulty at all about where you place an operating theatre. If you have an underground operating theatre, providing you are willing to face the ventilation expense, and such an operating theatre would involve a great deal of expense, there is no reason why it should not be perfectly satisfactory.

More and more surgeons are taking up the attitude that when they are operating they prefer artificial light, so this aspect of the matter should provide no difficulty.

*Q.* MR. A. J. WRIGHT, M.B., F.R.C.S. : Where equal opportunities exist, does Mr. Elcock favour vertical or horizontal development of hospitals ?

*A.* MR. ELCOCK : If you have a very large city hospital situated on a site—we suppose it might be in the centre of Bristol, which is, as far as I know, on rock foundation—you could build your hospital fifty storeys high at very great economy. There is no doubt at all that a vertical type building for a large hospital has great economic value over the long horizontal type, which also loses in its annual revenue on account of upkeep expenses. With the vertical type you have one foundation, one roof, one supply plan for heating and electricity, and so on. In all cases we are advising a vertical hospital, unless there are causes in connection with the treatment of the disease which make a vertical type unwise.

*Q.* DR. P. PHILLIPS : With a view to possible aerial bombardment, should hospitals be situated in centres of cities in future ?

*A.* MR. ELCOCK : If we are going to consider the question of building after this war is over to accommodate ourselves to the possibility of such insanity happening again, we deserve to have all we get, and it does not matter in the slightest degree how high we build or how prominent we make our buildings if they are going to be knocked down—and we shall deserve to have them knocked down with the people in them. I don't think there is any object in building if we, in this country, are satisfied to go on living after the war as we are living at present.

*Q.* MR. F. M. BURRIS, J.P. : The " W " plan was stated to allow the light to come in, whereas the plans of a " star " building of an American hospital, Mr. Elcock said, did not get the whole of the light. As a novice it seems that the set of the two buildings is very similar, and it would be very interesting to know how the light does get into the one and does not get into the other.

*A.* MR. ELCOCK : The only objection to the " star " plan is that the inner angles of the star come so close together that they prevent the sunlight getting to the whole of the building, whereas if the star is kept apart, as in the "W" plan, you get the light coming down in the centre of the "W."

*Q.* MR. KENNETH H. PRIDIE, M.B., F.R.C.S. : How has it come about that the excellence of the hospital is inversely proportionate to the value of the patient ? For instance, if you are a lunatic you get teak doors and solid gun-metal handles and knobs, and if you are a wage-earner you get deal doors and brass knobs.

*A.* MR. ELCOCK : In regard to the distinction between the poor patient and the mental patient—the mental patient has to have first-class fittings, and you would be surprised to know how many mental patients will sit for a whole day worrying with their fingers at a little bit of screw which happens to have a slot in it as compared with one which hasn't, and how many will want to hang themselves on any conveniently placed hooks. The mental hospital has to be fitted up with extra strong things and extra good wood, but it is certainly a mistake for any hospital not to use first-class materials and spend money in capital outlay. Always go in for good stuff in the beginning and you will find you will save money in administrative costs and upkeep.

*Q.* THE REV. H. J. MORGAN : Could the speaker define a little further the relationship between the hospital in the urban area and the hospital in the rural area, and the functions they play, because a hospital in a rural area still has to serve a proportion of the people in that area who could not get into the urban hospital ? You did mention acute cases being treated in their own rural hospitals, but there are other cases besides to be considered.

*A.* MR. ELCOCK : Hospitals in rural areas are to continue to function as hospitals for the district, plus the assistance and advice of a Central District Council. They will increase their functions because they will want to meet the desires of the medical students who will want to see how to attack country disease and illness. But in addition they will keep in touch with the Central Council by having numbers of patients sent to the healthy surroundings of the country for convalescence and special treatment, whereby they may empty the beds in the large urban hospitals for the more acute cases. The rural hospitals should go on and even increase their functions, and particularly assist in the treatment of chronic diseases.

### DISCUSSION.

ALDERMAN J. J. MILTON, J.P. (Chairman, Bristol Public Health Committee) : I have been intrigued with the lecture this afternoon on the future of hospital development in Bristol, and I may say that so far as the Health Clinic side of the lecture is concerned, one could fully agree with what Mr. Elcock has said. But I am rather concerned with his idea of the main hospitals being centred round the teaching centre. I am one of those individuals who think the welfare of the patient and consideration of the patient is the thing that comes first, and that we have to build up our structure around the welfare and good of the patient.

In the large towns and cities of this country, large townships are springing up on the outskirts of towns which previously existed, and in my opinion it is necessary to provide for the needs of the people in the growing townships outside these cities.

You have a very good example here in Bristol. You have many districts growing very swiftly, and which will grow much more swiftly still after the war. In the hospital that serves one area they have had to increase on account of the need and the wants of the district; they have had to open up new departments; they have had to put up increased accommodation for their nurses—largely on account of the needs and wants in the district.

I visualize that this will go on. You will have to meet the wants of the people in these areas and you will have to make provision for it, and that provision you make will not have to be any second-rate provision. It will have to be of equal standard to that of any provision in the important centres of the city, and therefore, there is one thing I should like to hear more about from Mr. Elcock: whether he has fully made up his mind that what he suggests is going to be the best thing for the patient and for the general welfare of the people.

I fully agree with him as to the development of health services, and preventive medicine has to play an important part, but so far as allocation of the main hospitals are concerned of bringing them all within a subscribed area, then I disagree.

Mr. H. Evans: In deference to my Chairman, I should like to agree with what he has said, but surely if you are going to have good doctors you have got to have good medical students, and therefore a good medical school, and every opportunity should be given to that medical school to get all the information it can from the hospital, which should be close to the University and the school itself.

Mr. G. D. Gordon Hake, R.W.A., F.R.I.B.A.: I should like to thank Mr. Elcock on behalf of the architectural students for his courtesy in directing certain of his remarks particularly to them. I am sure they very much appreciated it.

Mr. Elcock: I think, Mr. Chairman, a locally uneducated person like myself would be extremely inadvised to make any attempt at a dogmatic reply to the very well-informed Alderman Milton, who actually knows the district from A to Z, has lived here all his life, and plays such an important part, as I know very well, in the health work of the district. It would be quite foolish for me to state anything dogmatic about anything which requires specific knowledge and consideration of conditions in the district.

I agree with him entirely that the patient comes first, the patient comes second, the patient comes third. The welfare of the patient is the whole object, but the welfare of the patient is only secured—the real welfare—by the attention of first-class trained doctors and nursing staff.

The years of studentship pass very quickly. The time is very limited in which study can be given to these things, and it therefore seems to me that a great deal of thought should be given to the possibility of erecting the Medical Centre so that it might be very advantageously situated with regard to its relationship to the medical school.

Time should not be wasted in travelling from hospital to hospital, and it would appear to me that you have a remarkable opportunity in the centre of Bristol adjacent to the University Medical School. You have a remarkable opportunity of erecting, therefore, a building which could be a very fine school of medical teaching and at the same time ensure the real welfare of the patient.

THE REV. MORGAN : Mr. Chairman, forgive me for keeping you a moment, but would it be possible for this address to be printed ? There was so much of real value in it, particularly in the second part, that I am sure some of us would like to read it at our leisure.

PROFESSOR J. A. NIXON : We are in a position to publish the lecture with its illustrations, and if we may have the opportunity of doing so in our local medical journal, reprints would be available for all of us, if Mr. Elcock is willing. We could illustrate it amply.

May I say how delightful the lecture has been to the doctors here ? It is quite true that Mr. Elcock strayed a little bit from what you might think was the strict architect's approach to this subject, but he has justification. I think he has taken as his example Sir Christopher Wren, who, long before he planned the rebuilding of London, was working for the members of the Royal Society in Oxford, and was actually the first man in the world to make an intra-venous injection.

I think this mixture of medical study with architecture is quite justified in hospital planning, and I very much appreciate all that Mr. Elcock has been telling us.

THE CHAIRMAN (Alderman A. W. S. Burgess, J.P.) stated that Mr. Elcock had no objection to the report being published.

MR. EUSTACE H. BUTTON, R.W.A., F.R.I.B.A., in proposing a vote of thanks to the lecturer, spoke on the need for education in order to ensure active co-operation from the whole nation in post-war reconstruction, and of the importance of men like Mr. Elcock who can enthuse others with plans for the future.

The vote of thanks was seconded by Mr. A. J. Wright, who thanked Mr. Elcock for the freshness of his outlook.

The Chairman concluded the lecture by laying emphasis on the fact that in planning for the future no one should be too dogmatic in his ideas. One should, rather, be willing to consider the ideas of others, and he thought a little compromise on these ideas might result in a very useful post-war world. He said that he himself had a perfectly open mind on the proposed central hospital, and he suggested that that should be the general attitude at the present juncture.

## Figures and Tables

**No. 1. f1:**
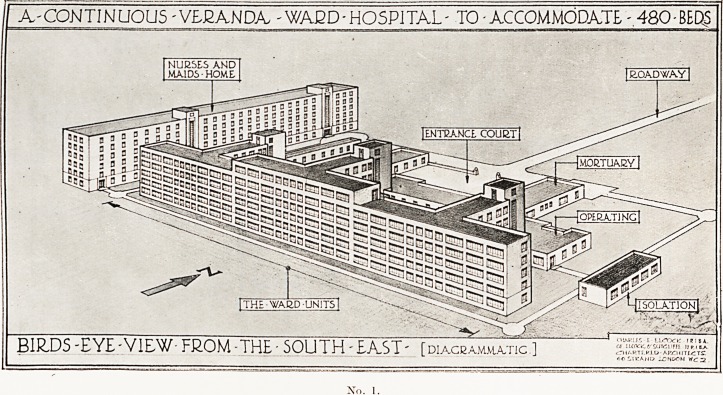


**No. 2. f2:**
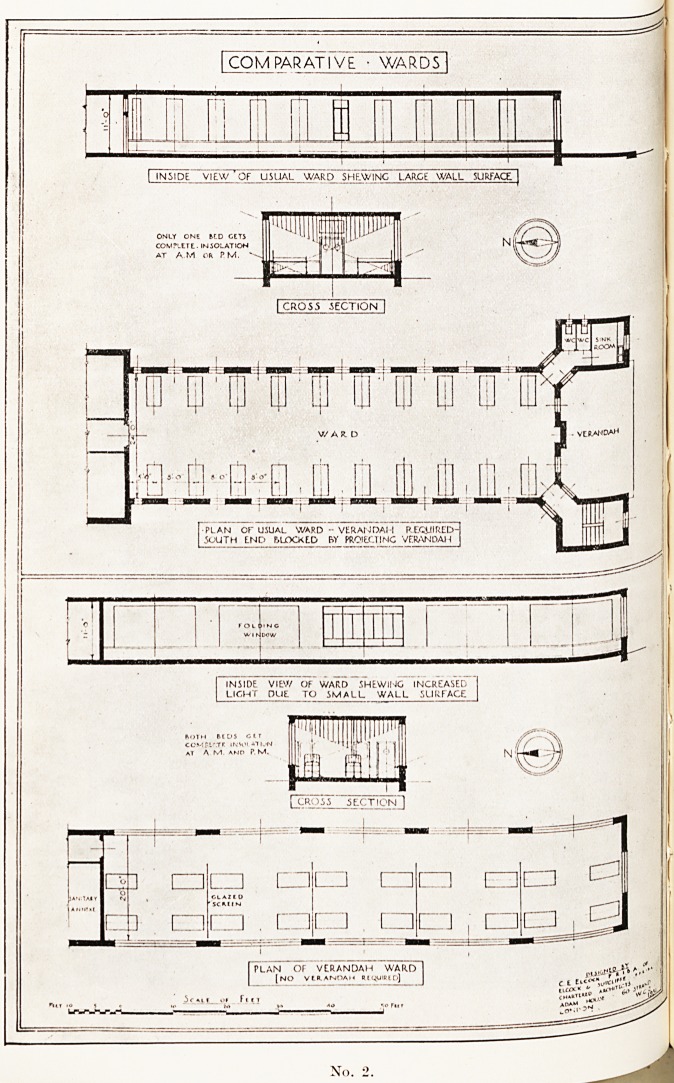


**No. 3. f3:**
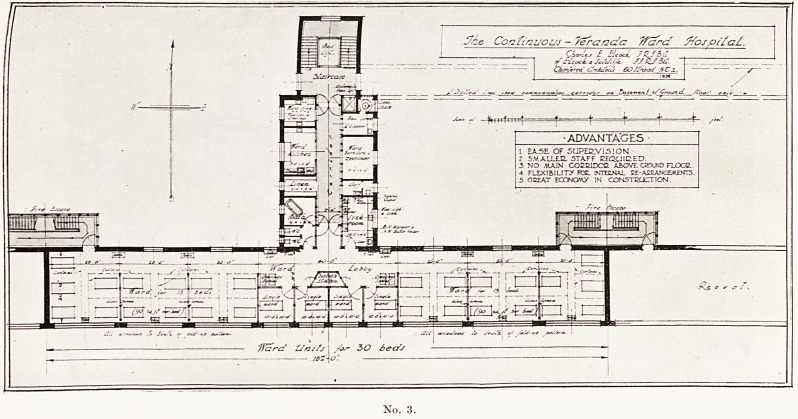


**No. 4. f4:**